# Outer dynein arm docking complex subunit 2 polymorphism rs7893462 modulates hepatocellular carcinoma susceptibility and can serve as an overall survival biomarker for hepatitis B virus-related hepatocellular carcinoma after hepatectomy: a cohort study with a long-term follow-up

**DOI:** 10.1186/s12957-023-03205-4

**Published:** 2023-10-13

**Authors:** Zhiming Zeng, Xiwen Liao, Ketuan Huang, Chuangye Han, Wei Qin, Hao Su, Xinping Ye, Chengkun Yang, Xin Zhou, Yongguang Wei, Shutian Mo, Junqi Liu, Chenlu Lan, Xinlei Huang, Zaida Huang, Kai Peng, Qiang Gao, Tao Peng, Guangzhi Zhu

**Affiliations:** 1 Department of Medical Oncology, The First Affiliated Hospital of Guangxi Medical University, Guangxi Zhuang Autonomous Region, Nanning, 530021 People’s Republic of China; 2 Department of Hepatobiliary Surgery, The First Affiliated Hospital of Guangxi Medical University, Guangxi Zhuang Autonomous Region, Nanning, 530021 People’s Republic of China; 3 Guangxi Key Laboratory of Enhanced Recovery After Surgery for Gastrointestinal Cancer, Guangxi Zhuang Autonomous Region, Nanning, 530021 People’s Republic of China

**Keywords:** rs7893462, ODAD2, HBV-related HCC, Overall survival, Hepatectomy

## Abstract

**Background:**

Genetic variants of outer dynein arm docking complex subunit 2 (ODAD2) have been reported to be closely associated with primary ciliary dyskinesia and colorectal cancer in previous studies, but the association of genetic variants of ODAD2 with hepatocellular carcinoma (HCC) has not been reported.

**Methods:**

We enrolled 80 healthy subjects and 468 Guangxi hepatitis B virus (HBV)-related HCC patients in this study. A case–control study method was used to explore the association of different ODAD2-rs7893462 genotypes with hepatocarcinogenesis. A comprehensive survival analysis was used to explore the association of rs7893462 with the prognosis of HBV-related HCC in Guangxi.

**Results:**

Through a case–control study, we observed that patients carrying the G allele of rs7893462 had a markedly increased susceptibility to hepatocarcinogenesis (odds ratio = 1.712, 95% confidence interval = 1.032–2.839, *P* = 0.037). We found that there were significant prognosis differences among three different genotypes of rs7893462. Nomogram analysis suggested that the contribution of rs7893462 polymorphisms to the prognosis of HBV-related HCC was second only to the BCLC stage. Stratified survival analysis suggested that the AG genotype of rs7893462 was an independent prognostic risk factor for HBV-related HCC. Joint effect survival analysis also observed that the AG genotype of rs7893462 combined with clinical parameters could significantly identify HBV-related HCC patients with different prognostic outcomes more accurately, and the AG genotype was also observed to be independent of clinical factors in HBV-related HCC survival.

**Conclusion:**

The ODAD2-rs7893462 polymorphisms can be used as an independent prognostic indicator of HBV-related HCC overall survival and are significantly associated with susceptibility to hepatocarcinogenesis.

**Supplementary Information:**

The online version contains supplementary material available at 10.1186/s12957-023-03205-4.

## Introduction

Liver cancer is a common solid tumor, and the most common pathological type is hepatocellular carcinoma (HCC) [1, 2]. Liver cancer has a high incidence in China, especially in Guangxi [3]. Hepatitis B virus (HBV) and aflatoxin are the most common risk factors for liver cancer in Guangxi [4–6]. Therefore, liver cancer is also the solid tumor with the highest mortality rate in Guangxi [7]. Numerous studies have shown that the occurrence of liver cancer is driven by both environmental and genetic factors [8]. We also preliminarily screened the genetic variants associated with the co-action of HBV and aflatoxin leading to hepatocarcinogenesis through multi-omics methods [9]. Our team’s previous research also found that a large number of single nucleotide polymorphisms (SNPs) are closely related to the genetic susceptibility of HCC, and some SNPs can also be used as prognostic biomarkers for HCC [10–16]. After screening in the previous preliminary research, we initially identified that outer dynein arm docking complex subunit 2 (ODAD2)-rs7893462 may be associated with the occurrence and prognosis of liver cancer. ODAD2 also known as armadillo repeat containing 4 (ARMC4). ODAD2 plays a certain function in spermatogenesis, and the deletion of its gene may affect male fertility [17]. Gao et al. reported a male patient with infertility due to the genetic phenotype of a compound heterozygous mutation in ODAD2 [18]. Case report shows infertility in PCD patients requiring improved sperm motility for conception [19]. Through a literature search, we also observed that the ODAD2 gene is markedly related to primary ciliary dyskinesia (PCD) and colorectal cancer (CRC) [20, 21]. The purpose of our current study was to evaluate the association of ODAD2-rs7893462 polymorphisms with overall survival of HCC based on our long-term follow-up Guangxi HBV-related HCC patients cohort, and its association with HCC susceptibility.

## Materials and methods

### Study subjects

We collected newly diagnosed HBV-related HCC patients who underwent hepatectomy at the Department of Hepatobiliary Surgery, The First Affiliated Hospital of Guangxi Medical University, from 2001 to 2013. The final follow-up date was set in January 2018. Our study was approved by the Ethics Committee of The First Affiliated Hospital of Guangxi Medical University; the approval number is NO.2022-KY-E-(218) and is in accordance with the Helsinki Declaration. Written informed consent was obtained from all patients for the procedures in this study. Genotyping of rs7893462 was obtained from the Human Exome Bead Chip 12v1-1 system (Illumina, Inc., San Diego, CA). All patients included in the Guangxi cohort were from the local Chinese population. All patients in the Guangxi cohort were positive for serum HBsAg. All patients with HBV-related HCC were newly diagnosed and confirmed by pathology. Patients’ demographic and clinical parameters were derived from the hospitalization records of the patients who were hospitalized. All patients were followed up every 3 months, mainly using telephone follow-up and outpatient visit records. Polymorphisms of rs7893462 in healthy subjects as the healthy control group were derived from the haplotype map (HAPMAP)—Han Chinese individuals in Beijing (CHB) cohort, which are freely available to the public through the database dbSNP (https://www.ncbi.nlm.nih.gov/projects/SNP/snp_ref.cgi?do_not_redirect&rs=rs7893462) [22–25].

### Comprehensive survival analysis

Association analysis of clinical parameters and rs7893462 genotypes in HBV-related HCC patients mainly relied on chi-square test and binary logistic regression. Through association analysis, we can understand that rs7893462 genotypes are closely related to those clinical parameters of HBV-related HCC, thus developing related genetic indicators for clinical parameters evaluation. We calculated the relationship between clinical parameters and prognosis of the Guangxi HBV-related HCC cohort by univariate Cox proportional risk regression model and log-rank test. Clinical parameters related to HBV-related HCC prognosis in Guangxi cohort were adjusted for inclusion in subsequent multivariate Cox proportional hazards regression models. We also used univariate and multivariate Cox proportional risk regression models to analyze the effects of different genetic patterns of rs7893462 on the prognosis of HBV-related HCC, so as to screen out risk alleles of rs7893462 associated with HBV-related HCC death. The stratified survival analysis was also performed in R4.0.2 using a multivariate Cox proportional hazards regression model, and the drawing of the forest plot for the stratified survival analysis was performed with the ggplot2 package. In the joint-effect survival analysis, the rs7893462 genotypes of different genetic patterns were combined with the clinical parameters related to HCC prognosis, so as to accurately evaluate the prognosis of patients in different subgroups.

### Statistical analysis

Association analysis of clinical parameters and rs7893462 genotypes in HBV-related HCC patients mainly relied on chi-square test and binary logistic regression. Odds ratio (OR) and 95% confidence intervals (CIs) were used to assess the risk ratio. Comparison of univariate survival analysis relied on the Kaplan–Meier method and log-rank test. Univariate and multivariate Cox proportional risk regression models were used to comprehensively compare survival differences among different subgroups. Hazard ratios (HRs) and 95% CIs were used to assess prognostic risk ratios. The drawing of the nomogram was performed on R4.0.2 using the rms package. Statistical calculations are performed on SPSS version 22.0. *P* value less than 0.05 considered the difference between the two groups to be statistically significant.

## Results

### Association analysis of clinical parameters and rs7893462 genotypes

A total of 80 healthy Asian subjects were recruited from the HAPMAP-CHB cohort. The genotype distribution of rs7893462 was as follows: 28 subjects with AA genotype, 38 subjects with AG genotype, and 14 subjects with GG genotype. A total of 468 HBV-related HCC patients from the Guangxi cohort were enrolled in this study, including 112 patients with the AA genotype, 212 patients with the AG genotype, and 144 patients with the GG genotype. Chi-square test comparison revealed significant differences in the distribution of rs7893462 three genotypes (AA, AG, and GG) between healthy subjects and HBV-related HCC patients in this study (*χ*^2^ = 7.521, *P* = 0.023). Through binary logistic regression analysis of rs7893462, we used AA genotype as the reference genotype and found that GG genotype can markedly increase the risk of HBV-related HCC (OR = 2.571, 95%CI = 1.293–5.114, *P* = 0.007), while no significant difference was observed between AG genotype and AA genotype (OR = 1.395, 95%CI = 0.813–2.391, *P* = 0.226). Then, when we combined the AG genotype and the GG genotype to compare with the AA genotype of rs7893462, we observed that there were also significant differences in the distribution of the AA genotype and the patients with the G allele (AG + GG) genotypes (*χ*^2^ = 4.400, *P* = 0.036). Binary logistic regression analysis suggests that patients with the G allele of rs7893462 have a significantly increased risk of HBV-related HCC compared to patients with the AA genotype (OR = 1.712, 95%CI = 1.032–2.839, *P* = 0.037). Then, when we combined the AG genotype and the AA genotype to compare with the GG genotype, we found that there were also significant differences in the distribution between the GG genotype and the patients with the A allele (AA + AG) genotypes of rs7893462 (*χ*^2^ = 5.863, *P* = 0.015). Binary logistic regression analysis suggests that patients with the A allele have a significantly reduced risk of HBV-related HCC compared to patients with the GG genotype of rs7893462 (OR = 0.477, 95%CI = 0.260–0.878, *P* = 0.017). Subsequently, we also compared homozygous (AA + GG) and heterozygous (AG) genotypes, and no significant difference in distribution was observed between homozygous and heterozygous genotypes of rs7893462 in this study cohort (*χ*^2^ = 0.133, *P* = 0.715; OR = 1.093, 95%CI = 0.679–1.757, *P* = 0.715).

### Comprehensive survival analysis

#### Survival analysis of demographic parameters in HBV-related HCC OS

We performed a survival analysis of clinical parameters to screen for prognostic indicators. In this study, we observed that radical resection is a clinical indicator related to the prognosis of HBV-related HCC, and patients who cannot obtain radical resection have a poor prognosis and can markedly increase the risk of death in HBV-related HCC patients (log-rank *P* = 0.044, HR = 1.168, 95%CI = 0.764–1.785, Supplementary Table S1). Tumor size greater than 5 cm is also a risk factor for clinical outcomes in HBV-related HCC (log-rank *P* < 0.0001, HR = 2.207, 95%CI = 1.639–2.973, Supplementary Table S1). In this study, the median survival time (MST) of HBV-related HCC with a tumor size larger than 5 cm was only 40 months, while the MST of HBV-related HCC patients with a tumor size less than 5 cm was 123 months (Supplementary Table S1). Similar phenomena can also be observed in patients with multifocal HBV-related HCC. The MST of patients with single-lesion HBV-related HCC was 68 months, while the MST of patients with multi-lesion HBV-related HCC was only 36 months (log-rank *P* = 0.004, HR = 1.151, 95%CI = 1.139–2.015, Supplementary Table S1). In this study, well-known HCC death-related risk factors such as portal vein tumor thrombus (PVTT, log-rank *P* < 0.0001, HR = 3.363, 95%CI = 2.494–4.535, Supplementary Table S1), α-fetoprotein (AFP, log-rank *P* = 0.041, HR = 1.334, 95%CI = 1.009–1.765, Supplementary Table S1), and Barcelona Clinic Liver Cancer (BCLC, log-rank *P* < 0.0001, B stage vs. A stage: HR = 1.769, 95%CI = 1.221–2.561; C stage vs. A stage: HR = 3.355, 95%CI = 2.486–4.527, Supplementary Table S1) stage were all observed to be markedly related to HBV-related HCC prognosis in this study. Based on the above results, six HBV-related and HCC-related prognostic indicators including tumor size, tumor number, radical resection, PVTT, BCLC stage, and serum AFP were finally incorporated into the subsequent multivariate Cox proportional hazards regression model for correction.

#### Survival analysis of rs7893462 polymorphisms in HBV-related HCC OS

**Fig. 1:**
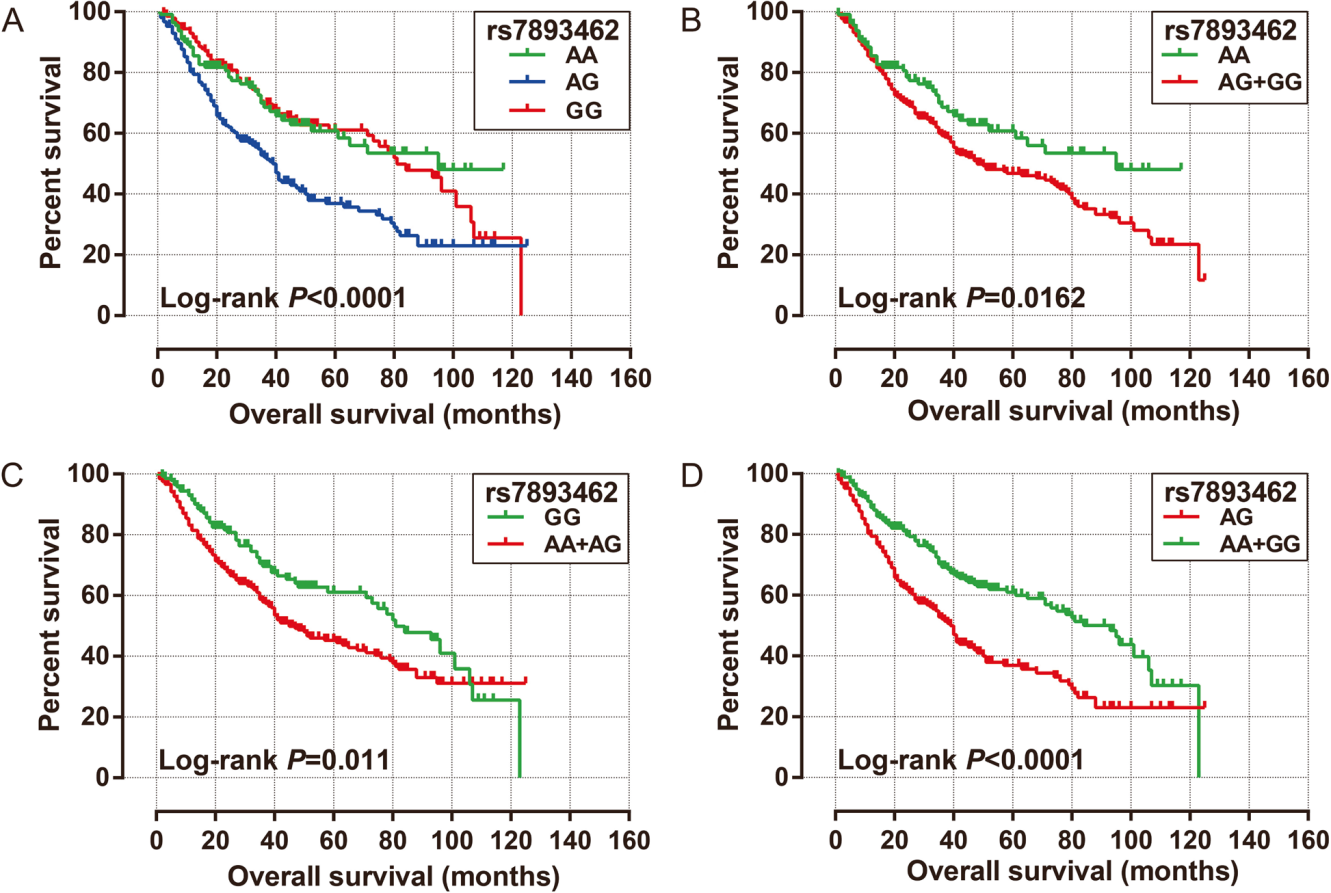
Univariate survival analysis curves of ODAD2-rs7893462 in HBV-related HCC overall survival time. **A** Kaplan–Meier curve of AA, AG, and GG; **B** Kaplan–Meier curve of AA and AG + GG; **C** Kaplan–Meier curve of GG and AA + AG; **D** Kaplan–Meier curve of AG and AA + GG

Since there are different genetic models for SNPs, we performed a survival analysis on these three genotypes of rs7893462 according to different genetic models. Compared with HBV-related HCC patients with AA genotype of rs7893462, patients with heterozygous AG genotype had a markedly worse prognosis (adjusted *P* = 0.002, adjusted HR = 1.806, 95% CI = 1.240–2.630, Table 1 and Fig. 1A). When we combined HCC patients with the G allele at rs7893462 into one group and compared them with the AA genotype, we did not observe a notable difference in prognosis between the two groups (adjusted *P* = 0.099, adjusted HR = 1.351, 95% CI = 0.945–1.932, Table 1, Fig. 1B). We combined HBV-related HCC patients with rs7893462 carrying the A allele (AA and AG genotypes) into one group, and compared them with GG genotype, and found that HBV-related HCC patients carrying the A genotype had a markedly increased risk of death (adjusted *P* = 0.002, adjusted HR = 1.652, 95% CI = 1.194–2.286, Table 1, Fig. 1C). When we compared the heterozygous AG genotype of rs7893462 as the reference group, we observed that the homozygous AA (adjusted *P* = 0.002, adjusted HR = 0.554, 95% CI = 0.380–0.807, Table 1) and GG (adjusted *P* < 0.0001, adjusted HR = 0.496, 95% CI = 0.352–0.698, Table 1) genotypes of rs7893462 were a protective factor significantly associated with prognosis in HBV-related HCC patients. After combining the HBV-related HCC patients with AA and GG genotypes of rs7893462 into one group, a significant protective effect was also observed by comparing them with heterozygous AG genotype (adjusted *P* < 0.0001, adjusted HR = 0.519, 95% CI = 0.389–0.693, Table 1, Fig. 1D).

**Table 1:** Survival analysis of ODAD2- rs7893462 in Guangxi HBV-related HCC patients

Genotype	Patients(*n* = 468)	MST (months)	Crude HR (95% CI)	Crude P	Adjusted HR (95% CI)	Adjusted *P*^ɠ^
**rs7893462**						
**AA**	112	95	1		1	
**AG**	212	39	1.916 (1.339–2.743)	< 0.0001	1.806 (1.240–2.630)	0.002
**GG**	144	81	1.059 (0.707–1.585)	0.781	0.895 (0.586–1.368)	0.61
**rs7893462**						
**AG**	212	39	1		1	
**AA**	112	95	0.522 (0.365–0.747)	0.0004	0.554 (0.380–0.807)	0.002
**GG**	144	81	0.553 (0.403–0.7577)	0.0002	0.496 (0.352–0.698)	< 0.0001
**AA + GG**	256	93	0.540 (0.413–0.705)	< 0.0001	0.519 (0.389–0.693)	< 0.0001
**rs7893462**						
**AA**	112	95	1		1	
**AG + GG**	356	48	1.515 (1.075–2.136)	0.018	1.351 (0.945–1.932)	0.099
**rs7893462**						
**GG**	144	81	1		1	
**AA + AG**	324	45	1.471 (1.088–1.988)	0.012	1.652 (1.194–2.286)	0.002

*Abbreviation*: *HBV* hepatitis B virus, *HCC* hepatocellular carcinoma, *HR* hazard ratio, *CI* confidence interval, *MST* median survival time, *OS* overall survival, *BCLC* Barcelona Clinic Liver Cancer, *AFP* α-fetoprotein, *NA* not available, *ODAD2* outer dynein arm docking complex subunit 2

^ɠ^OS adjusted for tumor number, tumor size, portal vein tumor thrombus, radical resection, BCLC, and AFP in multivariate Cox proportional risk regression model

#### Nomogram of rs7893462 polymorphisms in HBV-related HCC OS

**Fig. 2:**
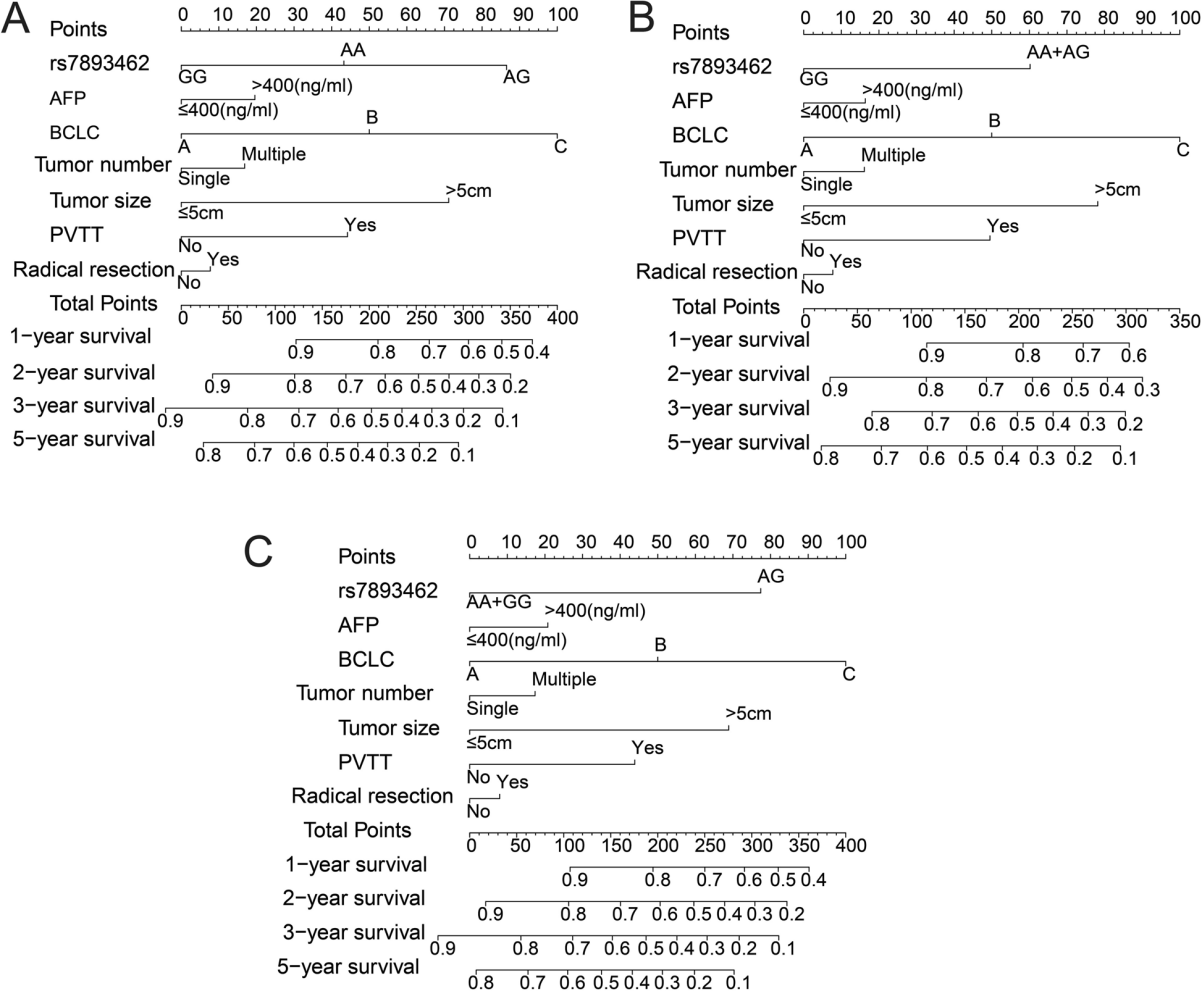
The nomogram of ODAD2-rs7893462 and clinical parameters in the Guangxi HBV-related HCC cohort. **A** Nomogram of AA, AG, and GG; **B** Nomogram of GG and AA + AG; **C** Nomogram of AG and AA + GG

To further understand the contribution of rs7893462 in the prognosis of HBV-related HCC, we also combined the prognosis-related indicators with rs7893462 to draw a nomogram that can individually predict patients’ prognosis. When we included the three genotypes of rs7893462 in the nomogram, we observed that the AG genotype was a risk factor for death of HBV-related HCC patients, and the contribution of rs7893462 polymorphisms in HBV-related HCC prognosis was second only to that of BCLC stage (Fig. 2A). After combining the HBV-related HCC patients with AA and AG genotypes of rs7893462 into one group, we observed that carrying the A allele is a risk factor for HCC patients, and its contribution to prognosis is second only to that of the BCLC stage. We speculated that the reason for this might be related to the greater contribution of the AG genotype to the prognosis of HCC (Fig. 2B). After combining the HBV-related HCC patients with AA and GG genotypes of rs7893462 into one group, we observed that the AG genotype had a significant impact on the clinical outcome of HCC patients in this study cohort, and its contribution was only second to that of BCLC stage (Fig. 2C).

#### Stratification analysis of rs7893462 polymorphisms in HBV-related HCC OS

**Fig. 3:**
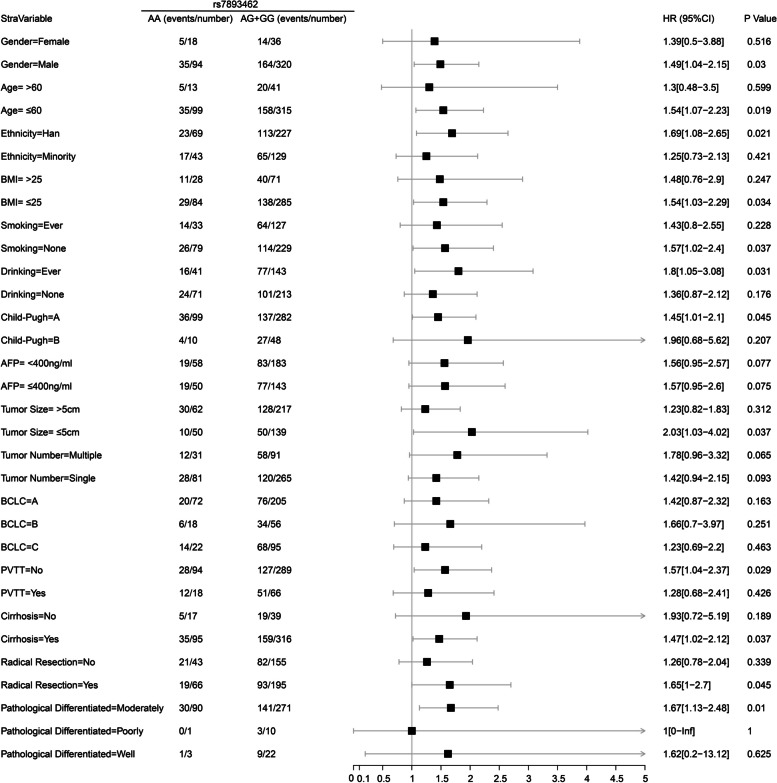
Forest plot of ODAD2-rs7893462 in Guangxi HBV-related HCC-stratified survival analysis between AA and AG + GG genotypes

**Fig. 4:**
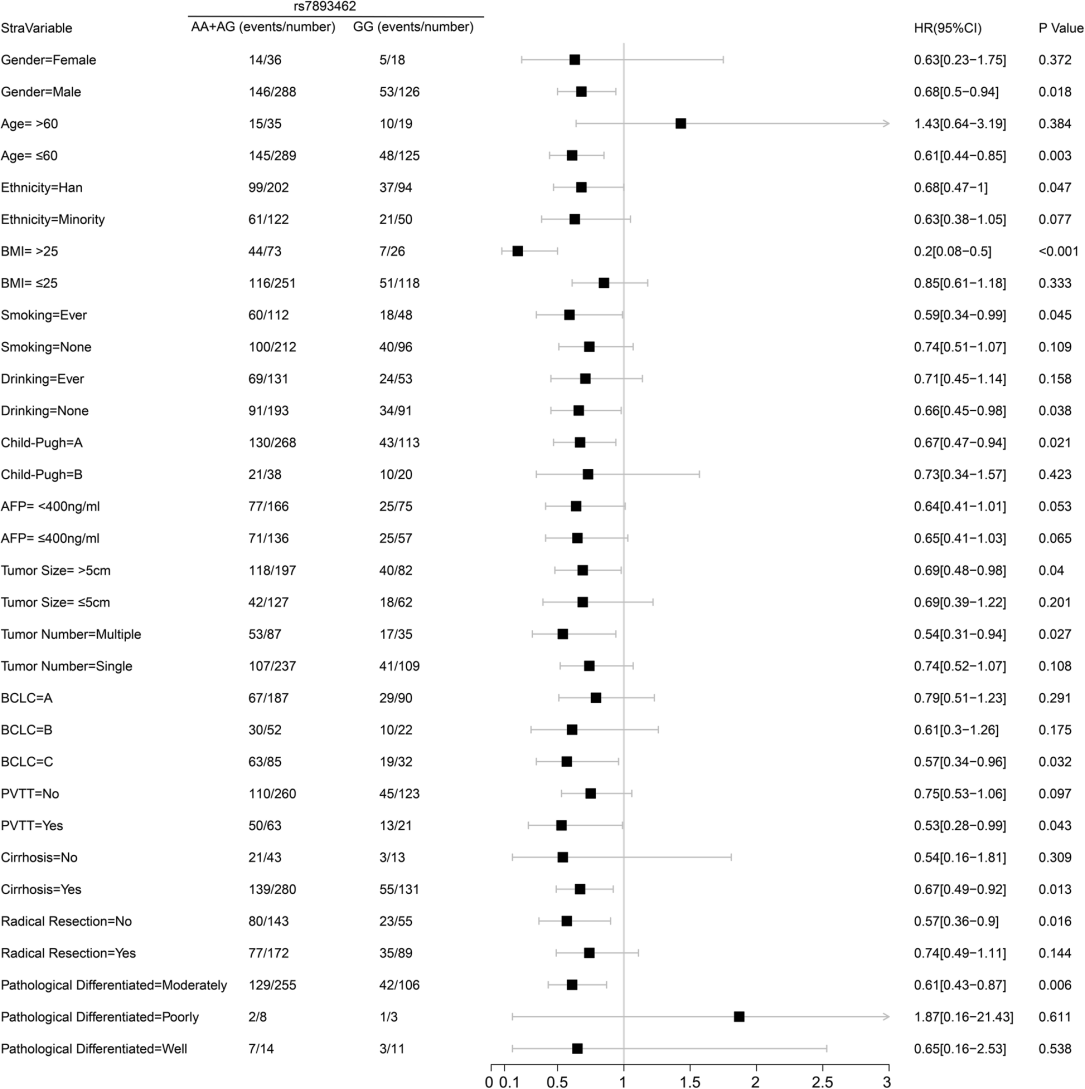
Forest plot of ODAD2-rs7893462 in Guangxi HBV-related HCC-stratified survival analysis between GG and AA + AG genotypes

**Fig. 5:**
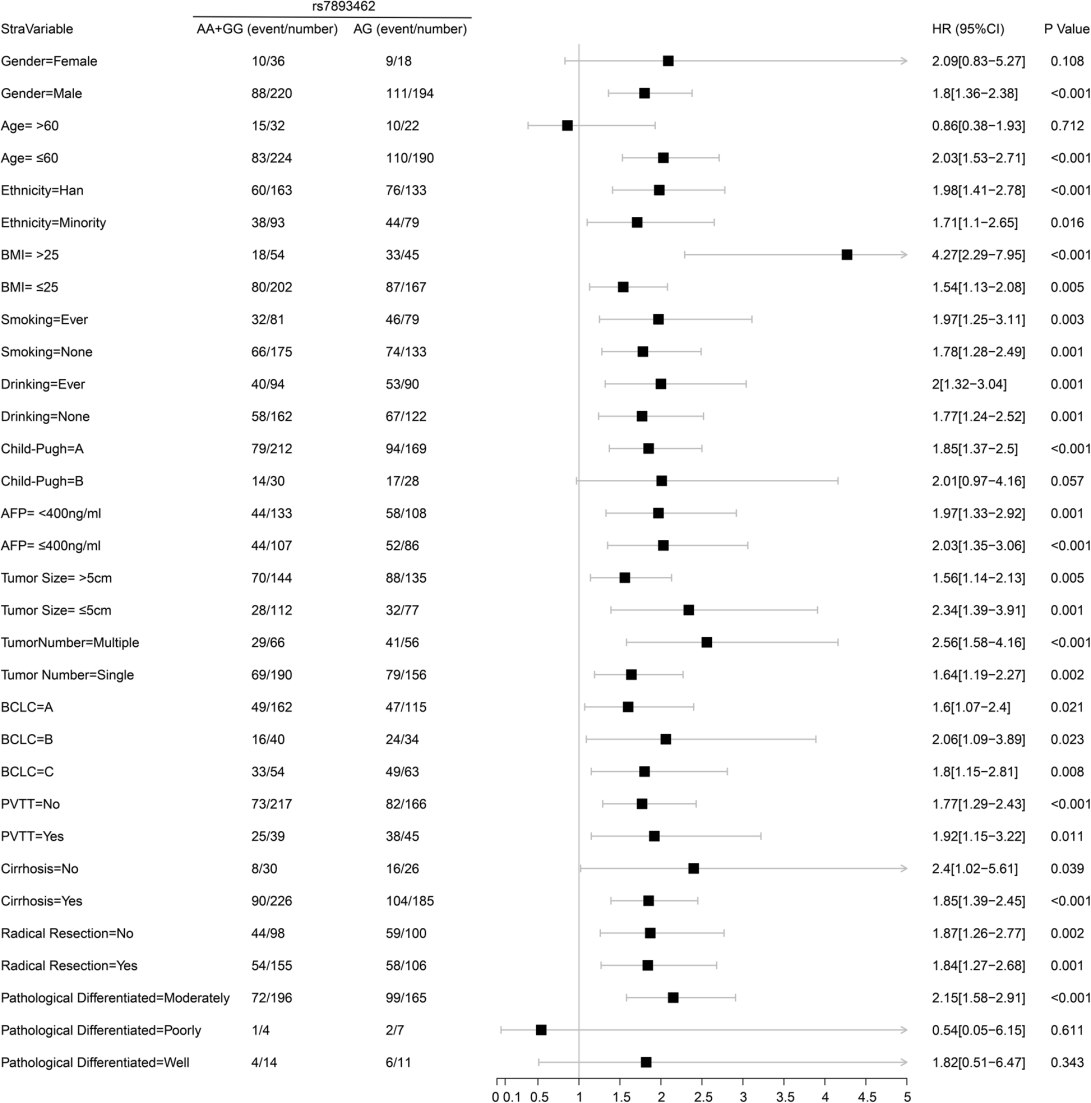
Forest plot of ODAD2-rs7893462 in Guangxi HBV-related HCC-stratified survival analysis between AG and AA + GG genotypes

In order to understand the prognostic differences of rs7893462 polymorphisms in different clinical subgroups, we conducted a stratified survival analysis of the multivariate Cox risk proportional regression model of rs7893462 to evaluate whether the prognostic differences of rs7893462 genotypes were independent of clinical factors. Under the grouping method of AA and AG + GG genotypes, we found significant differences in clinical outcomes between AA and AG + GG genotypes in 12 clinical HBV-related HCC subgroups. At the same time, the results of multivariate survival analysis suggested that patients with the G allele were a risk factor for poor prognosis in HBV-related HCC patients and may be a prognostic indicator independent of clinical parameters (Fig. 3). Under the grouping method of GG and AA + AG genotypes, we found significant differences in clinical outcomes between GG and AA + AG genotypes in 14 clinical HBV-related HCC subgroups. Paradoxically, our results of multivariate survival analysis suggested that patients with the GG genotype were a protective factor for the death of HBV-related HCC patients and may be a prognostic indicator independent of clinical parameters (Fig. 4). Under the grouping method of AG and AA + GG genotypes, we found significant differences in clinical outcomes between AG and AA + GG genotypes in all HBV-related HCC clinical subgroups except five clinical subgroups. The five clinical subgroups were female, older than 60 years, Child–Pugh B, and pathological graded poorly and well. At the same time, the results of multivariate survival analysis suggested that patients with AG genotype were an independent risk factor for poor prognosis in HBV-related HCC patients (Fig. 5). Through stratified survival analysis of three different SNP genetic models, we can infer that the AG genotype is an independent risk factor for death in HBV-related HCC patients.

#### Joint effect survival analysis of rs7893462 polymorphisms in HBV-related HCC OS

**Fig. 6:**
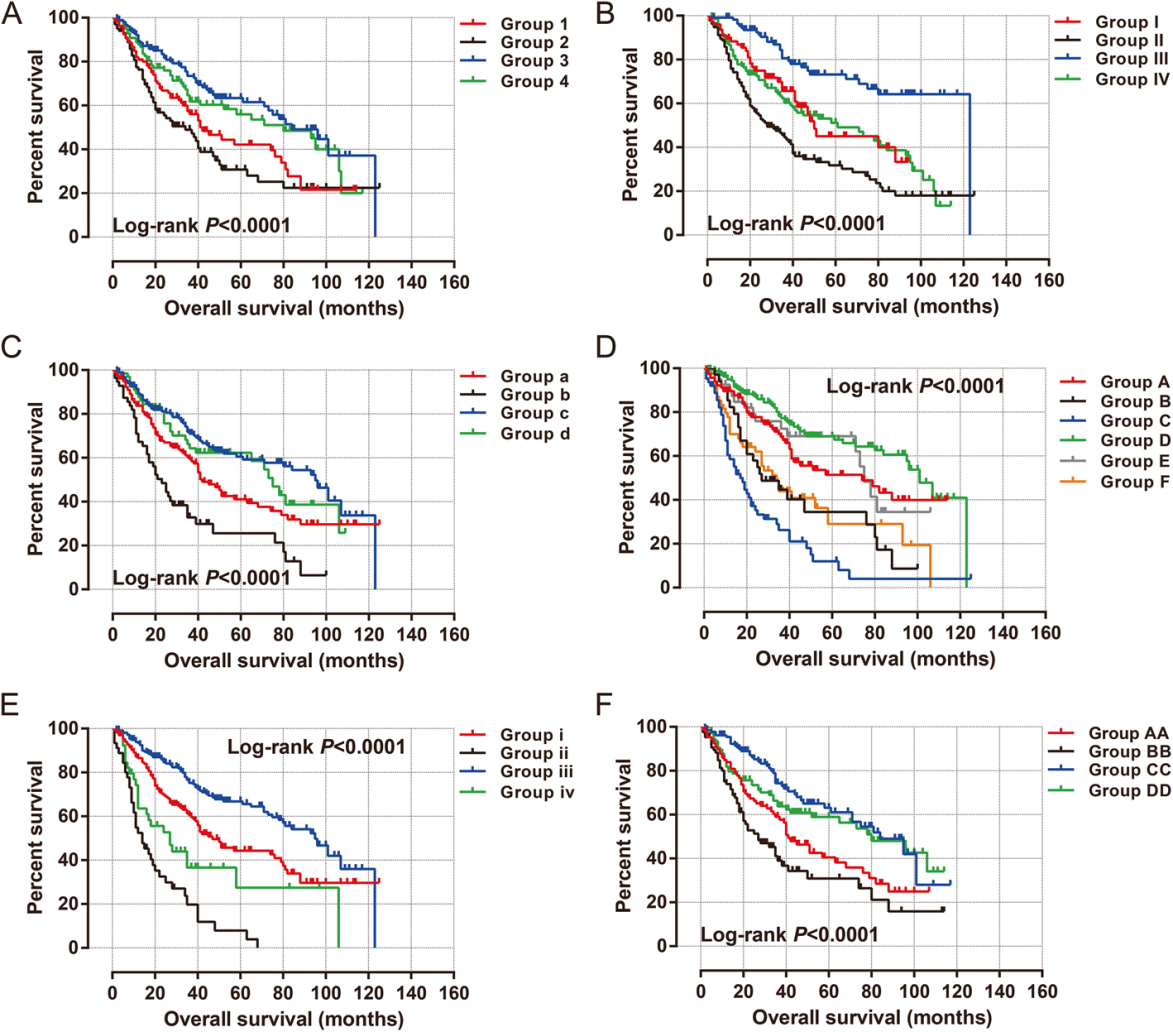
Joint effect survival analysis of ODAD2-rs7893462 and clinical parameters in Guangxi HBV-related HCC patients between AG and AA + GG genotypes. **A** Joint effect survival analysis of rs7893462 and Radical resection; **B** Joint effect survival analysis of rs7893462 and Tumor size; **C** Joint effect survival analysis of rs7893462 and tumor number; **D** Joint effect survival analysis of rs7893462 and BCLC; **E** Joint effect survival analysis of rs7893462 and PVTT; F Joint effect survival analysis of rs7893462 and serum AFP

**Fig. 7:**
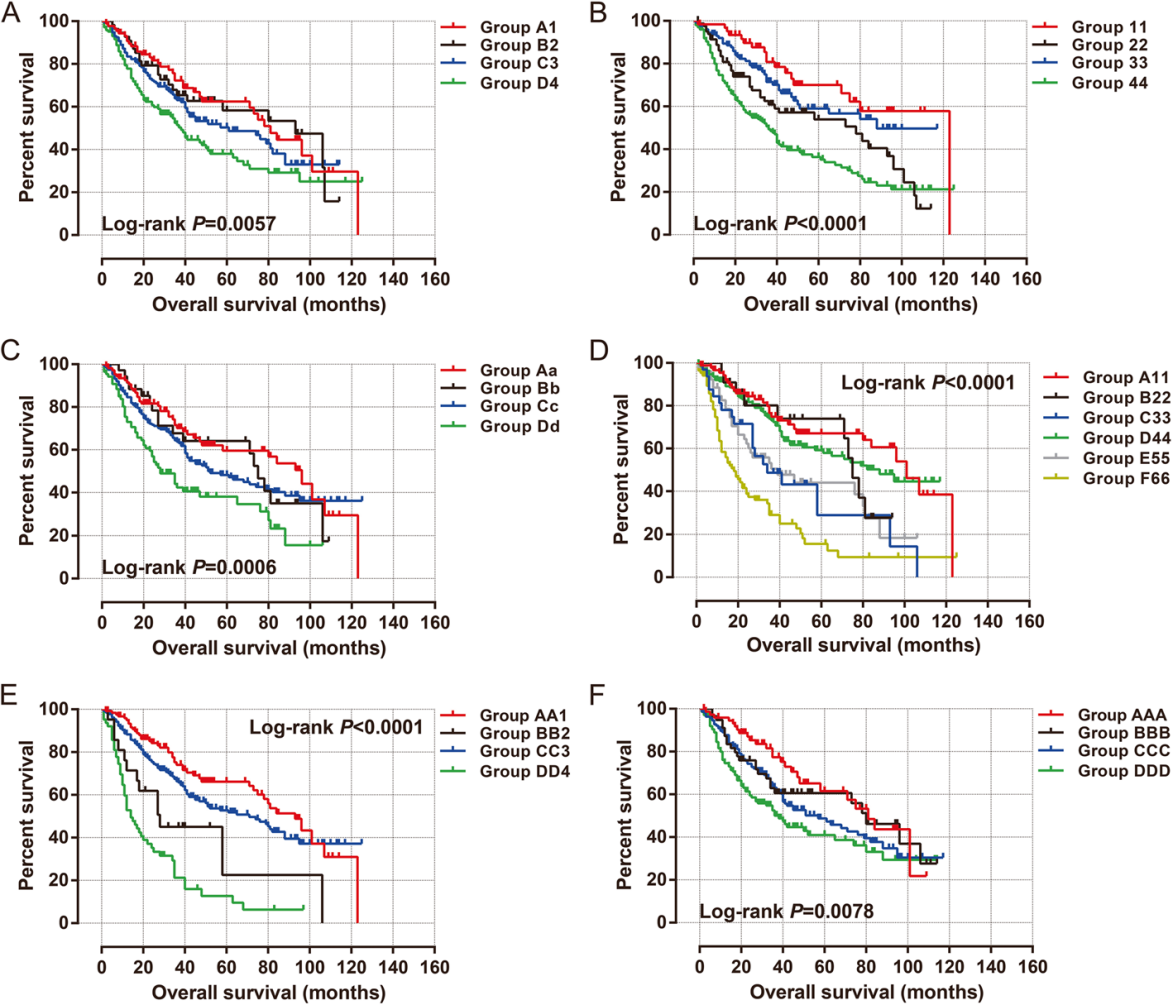
Joint effect survival analysis of ODAD2-rs7893462 and clinical parameters in Guangxi HBV-related HCC patients between GG and AA + AG genotypes. **A** Joint effect survival analysis of rs7893462 and radical resection; **B** Joint effect survival analysis of rs7893462 and tumor size; **C** Joint effect survival analysis of rs7893462 and tumor number; **D** Joint effect survival analysis of rs7893462 and BCLC; **E** Joint effect survival analysis of rs7893462 and PVTT; **F** Joint effect survival analysis of rs7893462 and serum AFP

After prognostic analysis, we found that patients with the AG genotype of rs7893462 had the worst prognosis among the three genotypes, so we combined the homozygous genotypes AA and GG into one group for survival analysis with the AG genotype. We then performed a joint effect survival analysis of the grouping mode of AG vs. AA + GG with six clinical parameters associated with the prognosis of HBV-related HCC. In univariate production analysis, we observed that combining this grouping pattern with clinical parameters could more accurately predict the prognosis of different subgroups of HBV-related HCC patients (all log-rank *P* < 0.0001, Fig. 6A–F). We performed a joint effect survival analysis of rs7893462 (AG vs. AA + GG) and six prognostic clinical parameters in the Guangxi HBV-related HCC cohort and observed that patients carrying the rs7893462 homozygous genotypes, regardless of whether they received radical resection, had a significantly better prognosis than patients with the AG genotype who underwent radical resection (Table 2). A similar phenomenon was also observed in the combined effect survival analysis of serum AFP and tumor number (Table 2). This may indicate that the AG genotype of rs7893462 may be an independent prognostic risk factor for HBV-related HCC. Subsequently, we also conducted a joint-effect survival analysis based on GG vs. AG + GG combined with clinical parameters (all log-rank *P* < 0.01, Fig. 7A–F). We observed that HCC patients carrying the A allele of rs7893462, regardless of single or multiple lesions, had a significantly unfavorable prognosis than GG genotype patients with single lesions (Table 3). We observed that patients with HCC carrying the A allele of rs7893462, regardless of whether they had PVTT or not, had a significantly worse prognosis than patients with the GG genotype with PVTT (Table 3).

**Table 2:** Joint effect survival analysis of ODAD2- rs7893462 (AG vs. AA + GG) and clinical parameters in Guangxi HBV-related HCC OS

Groups	Variables	Genotype	Patients(*n* = 468)	MST(months)	Crude HR (95% CI)	Crude *P*	Adjusted HR (95% CI)	Adjusted *P*
	**Radical resection**^**a**^	**rs7893462**						
**1**	Yes	AG	106	42	1		1	
**2**	No	AG	100	34	1.303 (0.906–1.875)	0.154	1.039 (0.695–1.555)	0.851
**3**	Yes	AA + GG	155	84	0.558 (0.385–0.808)	0.002	0.591 (0.397–0.881)	0.01
**4**	No	AA + GG	98	80	0.698 (0.472–1.034)	0.073	0.468 (0.300–0.731)	0.001
	**Tumor size**							
**I**	≤ 5 cm	AG	77	50	1		1	
**II**	> 5 cm	AG	135	28	1.754 (1.168–2.632)	0.007	1.557 (0.993–2.441)	0.054
**III**	≤ 5 cm	AA + GG	112	123	0.449 (0.270–0.747)	0.002	0.418 (0.240–0.727)	0.002
**IV**	> 5 cm	AA + GG	144	61	1.125 (0.740–1.711)	0.581	0.879 (0.555–1.393)	0.584
	**Tumor number**							
**a**	Single	AG	156	42	1		1	
**b**	Multiple	AG	56	23	1.930 (1.321–2.820)	0.001	1.536 (0.901–2.618)	0.115
**c**	Single	AA + GG	190	95	0.608 (0.440–0.841)	0.003	0.592 (0.417–0.841)	0.003
**d**	Multiple	AA + GG	66	75	0.750 (0.490–1.149)	0.187	0.610 (0.353–1.056)	0.077
	**BCLC**							
**A**	A	AG	115	74	1		1	
**B**	B	AG	34	27	2.010 (1.228–3.290)	0.005	1.575 (0.781–3.176)	0.204
**C**	C	AG	63	17	3.500 (2.331–5.254)	< 0.0001	3.037 (1.539–5.991)	0.001
**D**	A	AA + GG	162	101	0.634 (0.424–0.946)	0.026	0.647 (0.422–0.992)	0.046
**E**	B	AA + GG	40	75	0.925 (0.524–1.632)	0.788	0.706 (0.342–1.455)	0.345
**F**	C	AA + GG	54	34	1.906 (1.220–2.978)	0.005	1.296 (0.656–2.560)	0.455
	**Portal vein tumor thrombus**^**b**^							
**i**	No	AG	166	47	1		1	
**ii**	Yes	AG	45	14	3.598 (2.428–5.332)	< 0.0001	1.432 (0.762–2.692)	0.365
**iii**	No	AA + GG	217	95	0.570 (0.415–0.782)	0.0005	0.543 (0.387–0.761)	0.0004
**iv**	Yes	AA + GG	39	27	1.727 (1.102–2.706)	0.017	0.664 (0.335–1.314)	0.24
	**Serum AFP**^**c**^							
**AA**	AFP ≤ 400 (ng/mL)	AG	108	41	1		1	
**BB**	AFP > 400 (ng/mL)	AG	86	27	1.463 (1.004–2.131)	0.047	1.327 (0.900–1.957)	0.154
**CC**	AFP ≤ 400 (ng/mL)	AA + GG	133	84	0.518 (0.350–0.766)	0.001	0.589 (0.395–0.878)	0.009
**DD**	AFP > 400 (ng/mL)	AA + GG	107	80	0.675 (0.456–0.9996)	0.0498	0.604 (0.405–0.902)	0.014

*Abbreviation*: *HBV* hepatitis B virus, *HCC* hepatocellular carcinoma, *HR* hazard ratio, *CI* confidence interval, MST median survival time, *OS* overall survival, *BCLC* Barcelona Clinic Liver Cancer, *AFP* α-fetoprotein, *NA* not available, *ODAD2* outer dynein arm docking complex subunit 2

^a^Information of radical resection was unavailable in 9 patients

^b^Information of PVTT was unavailable in 1 patient

^c^Information of AFP was unavailable in 34 patients

**Table 3:** Joint effect survival analysis of ODAD2- rs7893462 (GG vs. AA + AG) and clinical parameters in Guangxi HBV-related HCC OS

Groups	Variables	Genotype	Patients(*n* = 468)	MST(months)	Crude HR (95% CI)	Crude *P*	Adjusted HR (95% CI)	Adjusted *P*
	**Radical resection**^**a**^	**rs7893462**						
**A1**	Yes	GG	89	81	1		1	
**B2**	No	GG	55	93	1.047 (0.619–1.773)	0.863	0.599 (0.327–1.094)	0.095
**C3**	Yes	AA + AG	172	61	1.318 (0.883–1.976)	0.177	1.277 (0.831–1.963)	0.265
**D4**	No	AA + AG	143	38	1.843 (1.238–2.744)	0.003	1.344 (0.858–2.108)	0.197
	**Tumor size**							
**11**	≤ 5 cm	GG	62	123	1		1	
**22**	> 5 cm	GG	82	78	2.093 (1.199–3.654)	0.009	1.619 (0.870–3.013)	0.128
**33**	≤ 5 cm	AA + AG	127	88	1.370 (0.788–2.382)	0.265	1.395 (0.764–2.549)	0.279
**44**	> 5 cm	AA + AG	197	36	3.032 (1.845–4.984)	< 0.0001	2.861 (1.650–4.959)	0.0002
	**Tumor number**							
**Aa**	Single	GG	109	96	1		1	
**Bb**	Multiple	GG	35	75	1.221 (0.693–2.151)	0.489	0.992 (0.508–1.934)	0.98
**Cc**	Single	AA + AG	237	52	1.350 (0.942–1.937)	0.102	1.487 (1.003–2.205)	0.048
**Dd**	Multiple	AA + AG	87	28	2.253 (1.495–3.395)	0.0001	2.014 (1.164–3.485)	0.012
	**BCLC**							
**A11**	A	GG	90	101	1		1	
**B22**	B	GG	22	75	1.435 (0.698–2.951)	0.326	1.209 (0.512–2.855)	0.665
**C33**	C	GG	32	34	2.614 (1.461–4.676)	0.001	1.710 (0.765–3.820)	0.191
**D44**	A	AA + AG	187	88	1.241 (0.802–1.921)	0.332	1.367 (0.846–2.209)	0.201
**E55**	B	AA + AG	52	39	2.388 (1.430–3.989)	0.001	1.829 (0.901–3.711)	0.095
**F66**	C	AA + AG	85	18	4.584 (2.935–7.161)	< 0.0001	3.691 (1.820–7.484)	0.0003
	**Portal vein tumor thrombus**^**b**^							
**AA1**	No	GG	123	93	1		1	
**BB2**	Yes	GG	21	28	2.569 (1.382–4.775)	0.003	1.038 (0.471–2.288)	0.926
**CC3**	No	AA + AG	260	71	1.336 (0.944–1.891)	0.102	1.474 (1.011–2.149)	0.043
**DD4**	Yes	AA + AG	63	14	4.886 (3.239–7.371)	< 0.0001	2.315 (1.239–4.327)	0.009
	**Serum AFP**^**c**^							
**AAA**	AFP ≤ 400 (ng/mL)	GG	75	81	1		1	
**BBB**	AFP > 400 (ng/mL)	GG	57	80	1.318 (0.756–2.296)	0.33	0.976 (0.555–1.716)	0.933
**CCC**	AFP ≤ 400 (ng/mL)	AA + AG	166	57	1.351 (0.975–2.405)	0.064	1.491 (0.944–2.353)	0.087
**DDD**	AFP > 400 (ng/mL)	AA + AG	136	38	2.080 (1.318–3.284)	0.002	1.783 (1.123–2.832)	0.014

*Abbreviation*: *HBV* hepatitis B virus, *HCC* hepatocellular carcinoma, *HR* hazard ratio, *CI* confidence interval, *MST* median survival time, *OS* overall survival, *BCLC* Barcelona Clinic Liver Cancer, *AFP* α-fetoprotein, *NA* not available, *ODAD2* outer dynein arm docking complex subunit 2

^a^Information of radical resection was unavailable in 9 patients

^b^Information of PVTT was unavailable in 1 patient

^c^Information of AFP was unavailable in 34 patients

#### Association analysis of clinical parameters and rs7893462 genotypes

Through survival analysis, we observed a significant difference in the clinical of the two subgroups between the AG genotype and the homozygous genotype, and we also observed the difference in prognosis between the GG and AG + AA genotypes in HBV-related HCC. To further understand the association of these two different genetic models with the clinical parameters of HCC, we performed an association analysis of different genetic models of rs7893462 with the clinical parameters, which are shown in Table 4. Under the grouping method of GG and AA + AG genotypes, we did not observe a significant association between the rs7893462 genotype and clinical parameters in the Guangxi HCC cohort (Table 4). Under the grouping method of AG and AA + GG genotypes, we also observed a significant association between the rs7893462 genotype and radical resection (*P* = 0.035, OR = 0.670, 95%CI = 0.462–0.973, Table 4). The same significant association can also be observed in HCC patients with BCLC A stage versus C stage (*P* = 0.025, OR = 0.608, 95%CI = 0.394–0.940, Table 4).

**Table 4:** Correlation analysis between ODAD2- rs7893462 and clinical parameters in Guangxi HBV-related HCC patients

Variables	GG (*n* = 144)	AA + AG (*n* = 324)	OR (95%CI)	*P*	AG (*n* = 212)	AA + GG (*n* = 256)	OR (95%CI)	*P*
**Age (years)**								
**≤ 60**	125	289	1		190	224	1	
**> 60**	19	35	0.797 (0.439–1.447)	0.46	22	32	1.234 (0.693–2.195)	0.475
**Gender**								
**Male**	126	288	1		194	220	1	
**Female**	18	36	0.875 (0.479–1.600)	0.66	18	36	1.764 (0.970–3.207)	0.063
**Ethnicity**								
**Han**	94	202	1		133	163	1	
**Minority**	50	122	1.135 (0.753–1.711)	0.54	79	93	0.961 (0.659–1.401)	0.834
**BMI**								
**≤ 25**	118	251	1		167	202	1	
**> 25**	26	73	1.320 (0.802–2.172)	0.28	45	54	0.992 (0.635–1.549)	0.972
**Smoking status**								
**None**	96	212	1		133	175	1	
**Ever**	48	112	1.057 (0.698–1.601)	0.8	79	81	0.779 (0.531–1.143)	0.202
**Drinking status**								
**None**	91	193	1		122	162	1	
**Ever**	53	131	1.165 (0.777–1.747)	0.46	90	94	0.787 (0.542–1.142)	0.206
**Child–Pugh**^**a**^								
**A**	113	268	1		169	212	1	
**B**	20	38	0.801 (0.447–1.437)	0.46	28	30	0.854 (0.491–1.485)	0.576
**Cirrhosis**^**b**^								
**No**	13	43	1		26	30	1	
**Yes**	131	280	0.646 (0.336–1.243)	0.19	185	226	1.059 (0.605–1.853)	0.842
**Radical resection**^**c**^								
**Yes**	89	172	1		106	155	1	
**No**	55	143	1.345 (0.899–2.013)	0.15	100	98	0.670 (0.462–0.973)	0.035
**Pathological diagnosis**^**d**^								
**Well differentiated**	11	14	1		11	14	1	
**Moderately differentiated**	106	255	1.890 (0.831–4.298)	0.13	165	196	0.933 (0.413–2.111)	0.868
**Poorly differentiated**	3	8	2.095 (0.447–9.814)	0.35	7	4	0.449 (0.104–1.934)	0.283
**Tumor size**								
**≤ 5 cm**	62	127	1		77	112	1	
**> 5 cm**	82	197	1.173 (0.788–1.747)	0.43	135	144	0.733 (0.505–1.065)	0.103
**Tumor number**								
**Single**	109	237	1		156	190	1	
**Multiple**	35	87	1.143 (0.727–1.799)	0.56	56	66	0.968 (0.639–1.464)	0.876
**BCLC**								
**A**	90	187	1		115	162	1	
**B**	22	52	1.138 (0.651–1.988)	0.65	34	40	0.835 (0.499–1.399)	0.494
**C**	32	85	1.278 (0.793–2.062)	0.31	63	54	0.608 (0.394–0.940)	0.025
**Portal vein tumor thrombus**^**e**^								
**No**	123	260	1		166	217	1	
**Yes**	21	63	1.419 (0.828–2.432)	0.2	45	39	0.663 (0.413–1.065)	0.089
**Serum AFP**^**f**^								
**AFP ≤ 400 (ng/mL)**	75	166	1		108	133	1	
**AFP > 400 (ng/mL)**	57	136	1.078 (0.714–1.628)	0.72	86	107	1.010 (0.690–1.479)	0.958

*Abbreviation*: *HBV* hepatitis B virus, *HCC* hepatocellular carcinoma, *HR* hazard ratio, *CI* confidence interval, *MST* median survival time, *BCLC* Barcelona Clinic Liver Cancer, *AFP* α-fetoprotein, *NA* not available, *BMI* body mass index

^a^Information of Child–Pugh was unavailable in 29 patients

^b^Information of cirrhosis was unavailable in 1 patient

^c^Information of radical resection was unavailable in 9 patients

^d^Information of pathological diagnosis was unavailable in 71 patients

^e^Information of PVTT was unavailable in 1 patient

^f^Information of AFP was unavailable in 34 patients

## Discussion

Previous studies have confirmed that SNP is involved in the regulation of susceptibility to liver cancer and may be markedly correlated with the prognosis of HCC patients. Zhang et al. conducted a case–control study using the genome-wide association study (GWAS) method and found that the 1P36.22 locus was closely related to the susceptibility of HBV-related HCC in the Chinese population [26]. Li et al. included HBV-related HCC TRIOS in a case–control study by the GWAS method and found that cyclin-dependent kinase 14 at 7Q21.13 (index rs10272859) was significantly associated with the susceptibility and prognosis of HBV-related HCC [27]. Jiang et al. also used the GWAS method to find that SNPs on STAT4 and HLA-DQ genes may be significantly correlated with HBV-related HCC susceptibility through large sample and multi-center methods, and they also found that the allele of the SNP locus of STAT4 was significantly correlated with its mRNA expression level [28]. We all know that there are differences in the allele frequencies of SNPs in different races and populations, so has the relationship between the susceptibility and prognosis of Guangxi HBV-HCC patients and SNPs been confirmed? Xue Qin and his research team used the Guangxi HBV-HCC population to study susceptibility and found that candidate SNPs of multiple genes were markedly associated with HBV-HCC susceptibility, and these SNPs may serve as potential markers in the Guangxi population [29–31]. Shan Li and his research team also found that SNPs in peptidyl-prolyl cis/trans isomerase NIMA-interacting 1 and tumor necrosis factor receptor superfamily 2 genes are associated with HBV-related HCC susceptibility [32, 33]. Qiu et al. revealed that damage-specific DNA-binding protein 2-rs1050244 is closely associated with HCC susceptibility through a case–control study of a large cohort of more than 2000 subjects [34]. Through the above literature review, we can understand that a large number of literature have reported candidate SNP sites related to HBV-related HCC susceptibility and prognosis, all of which have potential as HBV-related HCC biomarkers. In the present study, we identified a previously unreported candidate SNP that can be used as a prognostic biomarker in HBV-HCC patients and as an indicator for assessing susceptibility to hepatocarcinogenesis. Through a case–control study, we not only found that the G allele of rs7893462 was a risk factor for HBV-related HCC, but also found that the AG genotype of rs7893462 was an independent prognostic factor for HBV-related HCC in prognostic analysis, and the AG genotype can markedly increase the risk of death in HBV-HCC patients.

In previous studies, genetic variants of the ODAD2 gene have been extensively reported to be markedly related to PCD [21, 35–40]. However, its clinical application value in liver cancer has not been reported. Hjeij et al. used zebrafish and mice to verify that the ODAD2 gene is required for proper localization and anchoring of outer dynein arms [35]. Defects of this gene in the body can lead to the occurrence of PCD. Onoufriadis et al. found that genetic variation leading to deletion of the ODAD2 gene can result in loss of the distal external dynein arm motor responsible for ciliary beating, resulting in the immobility of cilia and ultimately PCD pathogenesis [36]. Raidt et al. used high-speed video-microscopy analysis technology to find a large number of PCD-related mutations. Among the genes with biallelic mutations finally determined, the allelic mutation of ODAD2 can be used to assist in the diagnosis of PCD [37]. Guan et al. used whole-exome technology to find a large number of genetic mutations associated with childhood PCD, and the ODAD2 gene was also observed to be mutated in these patients [38]. It has also been reported in the literature that ODAD2 is closely related to multiple cancers. Li et al. constructed a sarcoma prognostic signature containing the ODAD2 gene using RNA-sequencing data and alternative splicing data from The Cancer Genome Atlas cohort [41]. Liu et al. used the unit gamma measurement method to identify differentially expressed genes between nasopharyngeal carcinoma (NPC) and normal healthy nasopharyngeal tissues and then found that ODAD2 was differentially expressed in NPC tissues [42]. ODAD2 has also been reported to be closely associated with CRC. Liang et al. were the first to find that ODAD2 is frequently mutated in CRC tissues through exome sequencing analysis [20]. Martin et al. first identified ODAD2 as a novel negative regulator of nuclear factor-kappa B in CRC by validation-based insertional mutagenesis analysis. At the same time, the detection of clinical tissue expression showed that ODAD2 was significantly downregulated in tumor tissues, which may play a tumor suppressor role in CRC [43]. Zhang et al. revealed a number of novel biomarkers for peritoneal metastasis of gastric cancer based on whole-genome and transcriptome sequencing analysis technology, among which ODAD2 gene mutation may be closely related to the occurrence of peritoneal metastasis of gastric cancer [44]. Pongor et al. constructed a breast cancer mutation-related database based on 6697 patients and suggested that the mutation rate of ODAD2 in this cohort of breast cancers was greater than 5% [45].

Although there are some novel findings in our study, there are still some limitations in our study that need to be explained. First of all, this study was a single-center cohort study, lacking additional independent cohort validation. Secondly, our control group was healthy subjects, which would be more convincing if they were patients with chronic hepatitis B virus infection. Third, our HBV-HCC patients were followed up for up to 20 years, and the ultra-long follow-up time has certain limitations, which leads us to only obtain accurate overall survival, but cannot accurately obtain tumor-free survival time. Despite some research limitations, our findings can still provide a valuable prognostic indicator for HBV-related HCC patients.

## Conclusions

In conclusion, our study found that patients carrying the G allele of rs7893462 had a significantly increased susceptibility to hepatocarcinogenesis. Comprehensive survival analysis showed that rs7893462 was significantly associated with the prognosis of HBV-related HCC and may serve as a prognostic biomarker for HBV-related HCC after hepatectomy. Nomogram analysis suggested that the contribution of rs7893462 polymorphisms to the prognosis of HBV-related HCC was second only to the BCLC stage. Stratified and joint effect survival analysis suggested that the AG genotype of rs7893462 was an independent prognostic risk factor for HBV-related HCC. Since our study was a single-center cohort study, the results still need to be validated in future multi-center, large-cohort studies.

### Supplementary Information

**Additional file 1: Table S1.** Clinical parameters of HBV-related HCC patients in the First Affiliated Hospital of Guangxi Medical University cohort.

## Data Availability

All data generated or analyzed during this study are available from the corresponding author on reasonable request.

## References

[1] Llovet JM, Zucman-Rossi J, Pikarsky E, Sangro B, Schwartz M, Sherman M, Gores G (2016). Hepatocellular carcinoma. Nat Rev Dis Primers.

[2] Zhou J, Sun H, Wang Z, Cong W, Wang J, Zeng M, Zhou W, Bie P, Liu L, Wen T (2020). Guidelines for the diagnosis and treatment of hepatocellular carcinoma (2019 Edition). Liver Cancer.

[3] Chen W, Zheng R, Baade PD, Zhang S, Zeng H, Bray F, Jemal A, Yu XQ, He J (2016). Cancer statistics in China, 2015. CA Cancer J Clin.

[4] Qi LN, Bai T, Chen ZS, Wu FX, Chen YY, De Xiang B, Peng T, Han ZG, Li LQ (2015). The p53 mutation spectrum in hepatocellular carcinoma from Guangxi, China: role of chronic hepatitis B virus infection and aflatoxin B1 exposure. Liver Int.

[5] Long XD, Ma Y, Zhou YF, Ma AM, Fu GH (2010). Polymorphism in xeroderma pigmentosum complementation group C codon 939 and aflatoxin B1-related hepatocellular carcinoma in the Guangxi population. Hepatology.

[6] Xu L, Qian G, Tang L, Su J, Wang JS (2010). Genetic variations of hepatitis B virus and serum aflatoxin-lysine adduct on high risk of hepatocellular carcinoma in Southern Guangxi. China J Hepatol.

[7] Zeng H, Chen W, Zheng R, Zhang S, Ji JS, Zou X, Xia C, Sun K, Yang Z, Li H (2018). Changing cancer survival in China during 2003–15: a pooled analysis of 17 population-based cancer registries. Lancet Glob Health.

[8] Farazi PA, DePinho RA (2006). Hepatocellular carcinoma pathogenesis: from genes to environment. Nat Rev Cancer.

[9] Qi LN, Li LQ, Chen YY, Chen ZH, Bai T, Xiang BD, Qin X, Xiao KY, Peng MH, Liu ZM (2013). Genome-wide and differential proteomic analysis of hepatitis B virus and aflatoxin B1 related hepatocellular carcinoma in Guangxi, China. PLoS One.

[10] Liao X, Han C, Qin W, Liu X, Yu L, Lu S, Chen Z, Zhu G, Su H, Mo Z (2016). Genome-wide association study identified PLCE1- rs2797992 and EGFR- rs6950826 were associated with TP53 expression in the HBV-related hepatocellular carcinoma of Chinese patients in Guangxi. Am J Transl Res.

[11] Huang H, Liao X, Zhu G, Han C, Wang X, Yang C, Zhou X, Liang T, Huang K, Peng T (2022). Acyl-CoA binding domain containing 4 polymorphism rs4986172 and expression can serve as overall survival biomarkers for hepatitis B virus-related hepatocellular carcinoma patients after hepatectomy. Pharmgenomics Pers Med.

[12] Huang K, Liao X, Han C, Wang X, Yu T, Yang C, Liu X, Yu L, Chen Z, Qin W (2019). Genetic variants and expression of cytochrome p450 oxidoreductase predict postoperative survival in patients with hepatitis B virus-related hepatocellular carcinoma. J Cancer.

[13] Zhu G, Liao X, Han C, Liu X, Yu L, Qin W, Lu S, Su H, Chen Z, Liu Z (2017). ALDH1L1 variant rs2276724 and mRNA expression predict post-operative clinical outcomes and are associated with TP53 expression in HBV-related hepatocellular carcinoma. Oncol Rep.

[14] Yu L, Liu X, Han C, Lu S, Zhu G, Su H, Qi W, Liao X, Peng T (2016). XRCC1 rs25487 genetic variant and TP53 mutation at codon 249 predict clinical outcomes of hepatitis B virus-related hepatocellular carcinoma after hepatectomy: a cohort study for 10 years' follow up. Hepatol Res.

[15] Liu X, Yu L, Han C, Lu S, Zhu G, Su H, Qin W, Liao X, Peng T (2016). Polymorphisms of HLA-DQB1 predict survival of hepatitis B virus-related hepatocellular carcinoma patients receiving hepatic resection. Clin Res Hepatol Gastroenterol.

[16] Han C, Liao X, Qin W, Yu L, Liu X, Chen G, Liu Z, Lu S, Chen Z, Su H (2016). EGFR and SYNE2 are associated with p21 expression and SYNE2 variants predict post-operative clinical outcomes in HBV-related hepatocellular carcinoma. Sci Rep.

[17] Cheng W, Ip YT, Xu Z (2013). Gudu, an Armadillo repeat-containing protein, is required for spermatogenesis in Drosophila. Gene.

[18] Gao Y, Xu C, Tan Q, Shen Q, Wu H, Lv M, Li K, Tang D, Song B, Xu Y (2021). Case report: novel biallelic mutations in ARMC4 cause primary ciliary dyskinesia and male infertility in a Chinese family. Front Genet.

[19] Ozkavukcu S, Celik-Ozenci C, Konuk E, Atabekoglu C (2018). Live birth after Laser Assisted Viability Assessment (LAVA) to detect pentoxifylline resistant ejaculated immotile spermatozoa during ICSI in a couple with male Kartagener's syndrome. Reprod Biol Endocrinol.

[20] Liang Y, Jiang L, Zhong X, Hochwald SN, Wang Y, Huang L, Nie Q, Huang H, Xu JF (2019). Discovery of aberrant alteration of genome in colorectal cancer by exome sequencing. Am J Med Sci.

[21] Kilinc AA, Cebi MN, Ocak Z, Cokugras HC (2021). The relationship between genotype and phenotype in primary ciliary dyskinesia patients. Sisli Etfal Hastan Tip Bul.

[22] International HapMap C (2005). A haplotype map of the human genome. Nature.

[23] International HapMap C (2003). The international HapMap project. Nature.

[24] Rotimi C, Leppert M, Matsuda I, Zeng C, Zhang H, Adebamowo C, Ajayi I, Aniagwu T, Dixon M, Fukushima Y (2007). Community engagement and informed consent in the International HapMap project. Community Genet.

[25] International HapMap C (2004). Integrating ethics and science in the International HapMap Project. Nat Rev Genet.

[26] Zhang H, Zhai Y, Hu Z, Wu C, Qian J, Jia W, Ma F, Huang W, Yu L, Yue W (2010). Genome-wide association study identifies 1p36.22 as a new susceptibility locus for hepatocellular carcinoma in chronic hepatitis B virus carriers. Nat Genet.

[27] Li Y, Zhai Y, Song Q, Zhang H, Cao P, Ping J, Liu X, Guo B, Liu G, Song J (2018). Genome-wide association study identifies a new locus at 7q21.13 associated with hepatitis B virus-related hepatocellular carcinoma. Clin Cancer Res.

[28] Jiang DK, Sun J, Cao G, Liu Y, Lin D, Gao YZ, Ren WH, Long XD, Zhang H, Ma XP (2013). Genetic variants in STAT4 and HLA-DQ genes confer risk of hepatitis B virus-related hepatocellular carcinoma. Nat Genet.

[29] Li S, Deng Y, Chen ZP, Huang S, Liao XC, Lin LW, Li H, Peng T, Qin X, Zhao JM (2011). Genetic polymorphism of interleukin-16 influences susceptibility to HBV-related hepatocellular carcinoma in a Chinese population. Infect Genet Evol.

[30] Liu Y, Huang L, Lu Y, Xi XE, Huang XL, Lu Q, Huang X, Li S, Qin X (2015). Relationships between the Osteocalcin gene polymorphisms, serum osteocalcin levels, and hepatitis B virus-related hepatocellular carcinoma in a Chinese population. PLoS One.

[31] Luo J, Chen S, Wang J, Ou S, Zhang W, Liu Y, Qin Z, Xu J, Lu Q, Mo C (2019). Genetic polymorphisms in complement receptor 1 gene and its association with HBV-related liver disease: a case-control study. Gene.

[32] Ma L, Chen S, Mao X, Lu Y, Zhang X, Lao X, Qin X, Li S (2018). The association between TNFR gene polymorphisms and the risk of hepatitis B virus-related liver diseases in Chinese population. Sci Rep.

[33] Huang L, Mo Z, Lao X, Zhang X, Liu Y, Sui J, Qin X, Li S (2016). PIN1 genetic polymorphisms and the susceptibility of HBV-related hepatocellular carcinoma in a Guangxi population. Tumour Biol.

[34] Qiu M, Liu Y, Zhou Z, Jiang Y, Lin Q, Huo R, Liang X, Yu X, Zhou X, Yu H (2020). Association between single-nucleotide polymorphism in MicroRNA target site of DDB2 and risk of hepatocellular carcinoma in a Southern Chinese population. Biomed Res Int.

[35] Hjeij R, Lindstrand A, Francis R, Zariwala MA, Liu X, Li Y, Damerla R, Dougherty GW, Abouhamed M, Olbrich H (2013). ARMC4 mutations cause primary ciliary dyskinesia with randomization of left/right body asymmetry. Am J Hum Genet.

[36] Onoufriadis A, Shoemark A, Munye MM, James CT, Schmidts M, Patel M, Rosser EM, Bacchelli C, Beales PL, Scambler PJ (2014). Combined exome and whole-genome sequencing identifies mutations in ARMC4 as a cause of primary ciliary dyskinesia with defects in the outer dynein arm. J Med Genet.

[37] Raidt J, Wallmeier J, Hjeij R, Onnebrink JG, Pennekamp P, Loges NT, Olbrich H, Haffner K, Dougherty GW, Omran H, Werner C (2014). Ciliary beat pattern and frequency in genetic variants of primary ciliary dyskinesia. Eur Respir J.

[38] Guan Y, Yang H, Yao X, Xu H, Liu H, Tang X, Hao C, Zhang X, Zhao S, Ge W, Ni X (2021). Clinical and genetic spectrum of children with primary ciliary dyskinesia in China. Chest.

[39] Yi T, Sun H, Fu Y, Hao X, Sun L, Zhang Y, Han J, Gu X, Liu X, Guo Y (2022). Genetic and clinical features of heterotaxy in a prenatal cohort. Front Genet.

[40] Emiralioglu N, Taskiran EZ, Kosukcu C, Bilgic E, Atilla P, Kaya B, Gunaydin O, Yuzbasioglu A, Tugcu GD, Ademhan D (2020). Genotype and phenotype evaluation of patients with primary ciliary dyskinesia: first results from Turkey. Pediatr Pulmonol.

[41] Li H, Yang J, Yang G, Ren J, Meng Y, Qi P, Wang N (2021). Identification of prognostic alternative splicing events in sarcoma. Sci Rep.

[42] Liu C, Guo P, Zhou L, Wang Y, Tian S, Ding Y, Wu J, Zhu J, Wang Y (2021). A practical method to screen and identify functioning biomarkers in nasopharyngeal carcinoma. Sci Rep.

[43] Martin M, Mundade R, Hartley AV, Jiang G, Jin J, Sun S, Safa A, Sandusky G, Liu Y, Lu T (2022). Using VBIM technique to discover ARMC4/ODAD2 as a novel negative regulator of NF-kappaB and a new tumor suppressor in colorectal cancer. Int J Mol Sci.

[44] Zhang J, Huang JY, Chen YN, Yuan F, Zhang H, Yan FH, Wang MJ, Wang G, Su M, Lu G (2015). Whole genome and transcriptome sequencing of matched primary and peritoneal metastatic gastric carcinoma. Sci Rep.

[45] Pongor L, Kormos M, Hatzis C, Pusztai L, Szabo A, Gyorffy B (2015). A genome-wide approach to link genotype to clinical outcome by utilizing next generation sequencing and gene chip data of 6,697 breast cancer patients. Genome Med.

