# Supplementary material for: Outer dynein arm docking complex subunit 2 polymorphism rs7893462 modulates hepatocellular carcinoma susceptibility and can serve as an overall survival biomarker for hepatitis B virus-related hepatocellular carcinoma after hepatectomy: a cohort study with a long-term follow-up
Source: World J Surg Oncol. 2023 Oct 13;21:322. doi: 10.1186/s12957-023-03205-4 (PMC10571289; doi:10.1186/s12957-023-03205-4)
Supplement: Supplementary file 1 — Additional file 1: Table S1. Clinical parameters of HBV-related HCC patients in the First Affiliated Hospital of Guangxi Medical University cohort. [file 12957_2023_3205_MOESM1_ESM.pdf]

Table S1. Clinical parameters of HBV-related HCC patients in the First Affiliated Hospital of Guangxi Medical University cohort

Variables	Patients(n=468)	No. of events	MST(months)	HR (95% CI)	Log-rank P
**Age (years)**					0.542
⩽60	414	193	65	1	
>60	54	25	44	1.137(0.749-1.727)	
**Gender**					0.222
Male	414	199	58	1	
Female	54	19	80	0.748(0.467-1.198)	
**Ethnicity**					0.194
Han	296	136	71	1	
Minority	172	82	51	1.199(0.910-1.580)	
**BMI**					0.993
⩽25	369	167	61	1	
>25	99	51	57	1.001(0.731-1.372)	
**Smoking status**					0.214
None	308	140	71	1	
Ever	160	78	44	1.191(0.902-1.574)	
**Drinking status**					0.09
None	284	125	75	1	
Ever	184	93	48	1.260(0.963-1.650)	
**Child–Pugh §**					0.304
A	381	173	63	1	
B	58	31	39	1.220(0.832-1.789)	
**Cirrhosis £**					0.471
No	56	24	82	1	
Yes	411	194	57	1.168(0.764-1.785)	
**Radical resection ƀ**					0.044
Yes	261	112	75	1	

No	198	103	41	1.314(1.005-1.717)	
**Pathological diagnosis ƙ**					0.551
Well differentiated	25	10	79	1	
Moderately differentiated	361	171	57	1.223(0.646-2.315)	
Poorly differentiated	11	3	NA	0.724(0.199-2.632)	
**Tumor size**					<0.0001
≤5 cm	189	60	123	1	
>5 cm	279	158	40	2.207(1.639-2.973)	
**Tumor number**					0.004
Single	346	148	68	1	
Multiple	122	70	36	1.151(1.139-2.015)	
**BCLC**					<0.0001
A	277	96	96	1	
B	74	40	71	1.769(1.221-2.561)	
C	117	82	22	3.355(2.486-4.527)	
**portal vein tumor thrombus hʋ**					<0.0001
No	383	155	80	1	
Yes	84	63	18	3.363(2.494-4.535)	
**Serum AFP ƛ**					0.041
AFP≤400 (ng/mL)	241	102	68	1	
AFP >400 (ng/mL)	193	96	42	1.334(1.009-1.765)	

Notes: § Information of Child–Pugh was unavailable in 29 patients; £ Information of cirrhosis was unavailable in 1 patients; ƀ Information of radical resection was unavailable in 9 patients; ƙ Information of Pathological diagnosis was unavailable in 71 patients; hʋ Information of PVTT was unavailable in 1 patients; ƛ Information of AFP was unavailable in 34 patients.

Abbreviation: HBV, hepatitis B virus; HCC, hepatocellular carcinoma; HR, hazard ratio; CI, confidence interval; MST, median survival time; PVTT, portal vein tumor thrombus; BCLC, Barcelona Clinic Liver Cancer; AFP, α-fetoprotein; NA, not available, BMI, Body Mass Index.
